# Chronobiological Factors Influencing Glycemic Control and Birth Outcomes in Gestational Diabetes Mellitus

**DOI:** 10.3390/nu17010157

**Published:** 2024-12-31

**Authors:** Amalia Messika, Yoel Toledano, Eran Hadar, Riva Tauman, Oren Froy, Raanan Shamir

**Affiliations:** 1 Helen Schneider Hospital for Women, Rabin Medical Center, Petach Tikva 4941492, Israel; amalia.messika@mail.huji.ac.il (A.M.); toledanoyoel@gmail.com (Y.T.); eranha2@clalit.org.il (E.H.); 2 Faculty of Medical and Health Sciences, Tel Aviv University, Tel Aviv 6997801, Israel; tauman@tlvmc.gov.il (R.T.); raanan@shamirmd.com (R.S.); 3 Institute of Biochemistry, Food Science and Nutrition, The Robert H. Smith Faculty of Agriculture, Food and Environment, The Hebrew University of Jerusalem, Rehovot 7610001, Israel; 4 Sieratzki-Sagol Institute for Sleep Medicine, Tel Aviv Sourasky Medical Center, Tel Aviv 6492416, Israel; 5 Institute of Gastroenterology, Nutrition and Liver Disease (RS), Schneider Children’s Medical Center of Israel, Petach Tikva 4920235, Israel

**Keywords:** chrono-nutrition, chronobiological disorders, gestational diabetes mellitus, glycemic control, birth weight percentile

## Abstract

Background/Objectives: Studies have shown that chronobiological factors may adversely affect glycemic control in patients with type 2 diabetes mellitus. We assessed the association of chronobiological factors with glycemic control and neonatal birth weight in women with GDM. Methods: A prospective cohort study included 208 women aged 18–45 years with a singleton pregnancy who were randomly selected from among women undergoing follow-up for GDM at the Maternal-Fetal Medicine Unit of a tertiary medical center. Nutrition, sleep, and lifestyle patterns were assessed from onset of GDM until birth along with glycemic control and birth outcomes. Results: Multivariate analyses on a cohort of 208 women revealed that suboptimal glycemic control was associated with a late breakfast (RR = 2.26; 95% CI 1.09–4.67), increased carbohydrate intake in the evening (RR = 1.19; 95% CI 1.003–1.42), and poor sleep quality (RR = 2.14; 95% CI 1.04–4.41). The adjusted relative risk for neonatal birth weight above the 85th percentile was associated with increased carbohydrate intake in the morning (RR = 1.70; 95% CI 1.30–2.23) and increased carbohydrate intake in the evening (RR = 1.39; 95% CI 1.16–1.67). Conclusions: Chronobiological factors are associated with suboptimal glycemic control and birth weight above the 85th percentile in women with GDM. The study was registered under ClinicalTrials.gov.org, identifier: NCT02916667.

## 1. Introduction

Circadian rhythms, regulated by internal circadian clocks, are physiological and behavioral changes that follow a 24 h cycle. The suprachiasmatic nucleus (SCN) serves as the central clock, relaying signals to internal peripheral clocks in response to external light absorbed by the retina. The peripheral clocks are also affected by other factors, such as feeding regimens, food ingredients, sleeping schedule and patterns, physical activity, and social interactions. Dys-synchronization between the internal circadian rhythms and the external signals may lead to circadian rhythm disorders, also known as “chronobiological disorders” [1,2].

Chronobiological factors include sleep, chrono-nutrition, and chrono-obesity. Chrono-nutrition factors include eating schedules, eating patterns, and specific nutrients. An appropriate chrono-nutritional eating pattern includes timed meals—breakfast adjacent to waking up and dinner 12 h from waking up and not in the late evening or before bedtime [3,4,5]. Several studies have demonstrated that appropriate chrono-nutritional eating patterns have metabolic benefits on glycemic profile [6,7,8]. Chrono-obesity is defined as obesity that results from night-time eating, exposure to artificial light at night, and poor sleep quality [9,10].

Another circadian factor that affects metabolic control is chronotype. Several studies have demonstrated that the evening chronotype is associated with suboptimal glycemic balance relative to the morning chronotype [11]. Disorders of all these circadian factors are associated with an increased risk of metabolic syndrome as well as gestational diabetes mellitus (GDM) [12,13].

GDM is defined as glucose intolerance with the onset or first recognition during pregnancy. The prevalence of GDM ranges from 1% to 28% worldwide [14]. GDM is associated with adverse feto-maternal outcomes, including glycemic and obstetric complications. Older maternal age, obesity, history of GDM, sleep disorders, and familial diabetes have all been shown to increase the risk for GDM [15]. Recent studies have begun to explore the relationship between chronobiological factors and GDM. Our group conducted a randomized controlled trial investigating the impact of a chrono-nutritional and sleep hygiene intervention on maternal glycemic control and the proportion of birth weight for newborns being at/more than the 85th percentile in women with GDM. The intervention significantly reduced the proportion of women with suboptimal glycemic control, primarily due to decreased carbohydrate intake in the evening [16].

Furthermore, several studies have highlighted the potential impact of meal timing on glycemic control during pregnancy [17,18,19]. Despite these emerging studies, the relationship between chronobiological factors and GDM remains understudied.

The aim of this study was to assess the potential association of chronobiological factors with glycemic control and neonatal birth weight in women with GDM.

## 2. Materials and Methods

### 2.1. Design and Population

This was a prospective study. The cohort consisted of women aged 18–45 years with a singleton pregnancy who were diagnosed with GDM at 24–28 gestational week. Women were randomly selected from among those undergoing follow-up for GDM at the Maternal-Fetal Medicine Unit of Rabin Medical Center, a tertiary university-affiliated hospital, between 1 November 2016 and 31 December 2018. GDM was evaluated in a two-step procedure according to the criteria of Carpenter and Coustan [20]. Exclusion criteria were chronic medical conditions (preexisting diabetes, cardiovascular diseases, hypertension, hyperthyroidism), shift work, and intake of glucose-lowering medications before GDM diagnosis. At enrolment, all women signed an informed consent form to participate in the study. The study was conducted according to the principles of the Declaration of Helsinki and approved by the Institutional Review Board of Rabin Medical Center (approval No. 0277-15-RMC, May 2015). The study was registered under ClinicalTrials.gov.org, with identifier NCT02916667. All participants underwent a thorough interview that included information on chrono-nutrition, sleep, and chronotype.

### 2.2. Chrono-Nutrition Evaluation

Data on the time and quantity of daily food consumption (total calories and calories from proteins, carbohydrates, and fats) were collected in an interview, according to the Nutrition Surveys Unit (MABAT), Israel Ministry of Health, 2016 [21]. The data were adjusted to the individual reported sleep–wake times on the day previous to the interview, categorized as follows: wake-up to 12 noon (morning interval), 12 noon to 6 p.m. (afternoon interval), and 6 p.m. to bedtime (evening interval). Increased carbohydrate intake was defined as an increase of 10 g at each interval of the day. In addition, the time intervals between wake-up to breakfast, and between the last meal to bedtime, were recorded and categorized into up to 30 min, 30–60 min, 60–120 min, or > 120 min.

### 2.3. Sleep Evaluation

Sleep quality was assessed using the validated diagnostic Pittsburgh Sleep Quality Index (PSQI) [22]. Excessive daytime sleepiness was assessed using the validated Epworth Sleepiness Scale (ESS) [23]. In addition, participants were asked to report the frequency of snoring before and during pregnancy, as follows: never, 1–2 times per month, 1–2 times per week, 3–4 times per week, or almost every day.

### 2.4. Outcome Measures

The primary outcome measures of the present study were maternal glycemic control and neonatal birth-weight percentile.

Glycemic control: Patients were instructed to self-monitor their blood glucose (SMBG) using a glucometer. At the beginning of the follow-up period, they were told to measure glucose level 6 times daily (fasting, pre-prandial, and 1 or 2 h post-prandial). The frequency was reduced to 4 times daily (fasting and post-prandial) when target capillary blood glucose levels were met: <95 mg/dL during fasting, <100 mg/dL before meals, <140 mg/dL 1 h after meals, and <120 mg/dL 2 h after meals. Glycemic control was considered good when at least 80% of the capillary glucose measurements were below target, and suboptimal when fewer than 80% were at target [24].

Neonatal birth-weight percentile: neonates with risk of overweight were defined as birth weight at or above the 85th percentile according to WHO gender-specific growth charts [25] and population-based centiles of standards of birth weight of live-born infants in Israel [26].

### 2.5. Statistical Analysis

Sample size was calculated based on the standard deviation of the primary outcome derived from publications of the National Surveys Unit (infant MABAT) of the Israel Ministry of Health, assuming a two-sided test with 80% statistical power and a 5% significance level. Prior to conducting statistical analyses, we assessed the normality of continuous variables using the Shapiro–Wilk test and visual inspection of Q-Q plots. For continuous variables that were not normally distributed, we applied appropriate transformations (log transformation and square root transformation) to achieve normality when possible. In cases where transformations did not result in a normal distribution, we used non-parametric tests for analysis. Numeric variables are reported as mean and standard deviation, and categorical variables are reported as number and percent. Proportions were compared between groups with univariate analysis. Between-group comparisons were performed with a chi-square test (when there were few observations) or Fisher’s exact test for categorical values; the Wilcoxon signed-rank test for categorical variables with an asymmetric distribution; and a paired-sample *t*-test for continuous variables. All tests were two-sided; a *p* value of ≤0.05 was considered significant. To examine possible relationships between the dependent variables (study outcomes) and the independent variables, a multivariate analysis was performed, and the variables found to be significant on univariate analysis or that had high medical importance in terms of the primary outcomes were entered into a logistic regression model. The relative risk and 95% confidence interval (CI) were calculated; a *p* value of <0.05 was considered significant. All data were generated with SPSS software, version 24.0 (IBM International, Armonk, NY, USA).

## 3. Results

**Figure 1:**
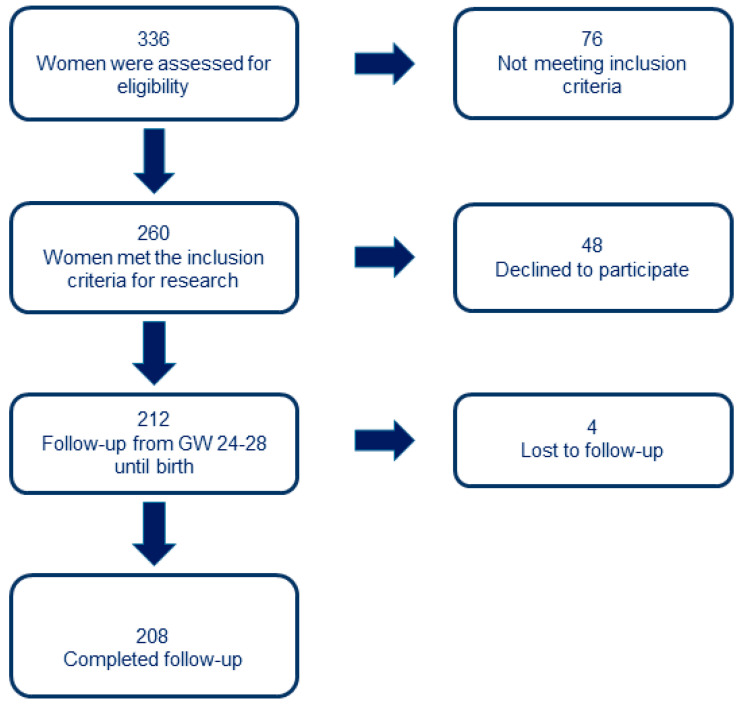
Study flowchart.

A total of 336 women were assessed for eligibility. Of those, 76 did not meet inclusion criteria, and 48 declined to participate. In the end, 212 women agreed to participate in the study. Among them, 3 women were lost to follow-up, and one pregnancy ended in stillbirth due to a chromosomal defect. The final cohort consisted of 208 women (Figure 1).

Demographic, anthropometric, and glycemic characteristics of study participants at time of GDM diagnosis are presented in Table 1. A total of 29.3% of the women had a pre-pregnancy BMI in the overweight range (25.0–29.9 kg/m^2^), while 29.8% were classified as obese (BMI ≥ 30.0 kg/m^2^). A family history of type 2 diabetes in first-degree relatives was reported by 38.9% of the women. Among the 136 women who had previously given birth, 33% reported a history of GDM, and 37.5% had at least one large-for-gestational-age (LGA) newborn in their prior pregnancies.

Lifestyle, sleep, and chronobiological characteristics are presented in Table 2. A total of 17.3% of women reported smoking prior to pregnancy, with 5.3% continuing to smoke during pregnancy. Current physical activity was reported by 21.1% of women. Furthermore, coffee consumption was notable, with 14% of the cohort consuming 3–4 cups per day, and 11.6% continuing to drink coffee after 5 pm. Poor sleep quality was reported by 45.2% of women, daytime sleepiness by 52.9%, and snoring by 22%. Additionally, 28.4% identified themselves as being an evening chronotype.

The total caloric intake and macronutrient content, as well as their distribution according to the time of food consumption (morning: wake-up–12 p.m.; afternoon: 12 p.m.–6 p.m.; evening: 6 p.m. to bed time) and time of breakfast relative to waking or the interval between the last meal and sleep onset, are presented in Table 3. The percentages of carbohydrate, protein, and fat intake in the morning were 48 ± 12, 18 ± 5, and 33 ± 13, respectively. The percentages of carbohydrate, protein, and fat intake in the evening were 36 ± 12, 20 ± 8, and 42 ± 12, respectively. The rate of late breakfast eaters (more than 30 min from wake-up to breakfast) was 75.5%, and the rate of late night-time eaters was 17.3% (fewer than 30 min from the last meal to bedtime).

Maternal glycemic, anthropometric, and pregnancy outcomes are presented in Table 4. Among 201 women evaluated for gestational weight gain according to Institute of Medicine (IOM) guidelines, 17.9% exceeded the recommended range of weight gain. For glycemic balance, 24.5% of the cohort required a combination of nutritional intervention and oral drug therapy, while 20.7% necessitated nutritional management supplemented with insulin therapy.

The rate of suboptimal glycemic control was 42.7%, and 19.2% of neonates had a birth weight at or above the 85th percentile.

Multivariate analysis revealed that in addition to treatment for glycemic control, late breakfast (RR = 2.26; 95% CI 1.09–4.67), excessive of a 10 g increase in carbohydrate intake in the evening interval (RR = 1.19; 95% CI 1.003–1.42), and poor sleep quality (RR = 2.14; 95% CI 1.04–4.41) were associated with increased risk for suboptimal glycemic control in women with GDM (Table 5).

Multivariate analyses were adjusted for maternal age, number of children, past birth-weight percentile for gestational age, treatment for glycemic balance, level of glycemic control, gestational weight gain, carbohydrate intake in the morning and/or evening interval, and sleep quality score. The results demonstrated that the maternal factors independently associated with birth weight at or above the 85th percentile were excessive carbohydrate intake during the morning interval (RR = 1.70; 95% CI 1.30–2.23) and during the evening interval (RR = 1.39; 95% CI 1.16–1.67) (Table 6). Significant findings were also noted for other factors known to be associated with birth weight at or above the 85th percentile in women with GDM, such as past birth weight at or above the 85th percentile (RR = 5.36; 95% CI 1.92–14.92) (Table 6).

## 4. Discussion

Our main aim was to investigate the effect of multiple chronobiological factors on glycemic control and neonatal birth weight in women with GDM. The main findings of this prospective study in women with GDM are that chronobiological factors are associated with glycemic control and neonatal birth weight. Specifically, a long interval between wake-up time and breakfast time, excessive carbohydrate intake in the evening, and poor sleep quality were all found to increase the risk of suboptimal glycemic control. In addition, excessive carbohydrate intake in the morning and in the evening were shown to increase the risk of birth weight above the 85th percentile.

These findings collectively suggest that both the content and timing of eating are crucial for diurnal glycemic control and birth outcomes in GDM. Previous studies on populations with type 2 diabetes or overweight/obesity have shown that a large carbohydrate-rich breakfast and a small low-carbohydrate dinner were associated with good glycemic control and weight management [27,28,29]. A systematic review by Gómez-Ruiz et al. concluded that restricting feeding to 2 to 3 meals per day and practicing time-restricted feeding with less than 10 h of daily food intake promote weight loss and glycemic control in patients with T2DM [30].

Several studies have explored the relationship between meal timing, fasting duration, and the distribution of energy and carbohydrate intake on metabolic health during gestation, revealing important insights into the management of GDM and overall maternal health. A randomized crossover trial conducted by Sacks et al. in women with gestational diabetes mellitus compared the effects of morning (07:00) and night (21:00) test meals. Despite having a lower carbohydrate content, the night meal resulted in a higher post-prandial glycemic response 3 to 9 h after consumption compared to the morning meal [17]. Similarly, Peterson et al. found that the correlation between carbohydrate intake and 1 h post-prandial glucose concentration was strongest for dinner, followed by lunch and breakfast [18]. Rasmussen et al. conducted a randomized crossover trial in normal-weight patients with GDM to further examine the link between 24 h energy/carbohydrate distribution and glycemic control. Participants followed two 4 day diets: one with high carbohydrate intake in the morning and another with high carbohydrate intake in the evening. The study found that high energy/carbohydrate intake in the morning led to lower fasting plasma glucose compared to high intake in the evening (83 vs. 92 mg/dL) [19]. Our recent randomized controlled trial found that chrono-nutritional and sleep hygiene intervention significantly improved maternal glycemic control in women with GDM. The intervention reduced the proportion of women with suboptimal glycemic control, with a relative risk of 0.28 (95% CI: 0.18–0.81) after adjusting for confounding factors. Notably, this improvement was primarily attributed to decreased carbohydrate intake during the evening (RR: 0.8, 95% CI: 0.64–0.99) [16].

Apart from the effect of meal timing and fasting duration, accumulating evidence supports that the distribution of energy and carbohydrates during the day may have important metabolic implications. However, the specific role of energy and carbohydrate distribution throughout the day on metabolic health during pregnancy remains unclear, with only a small number of studies investigating this relationship [31,32,33].

Current guidelines for women with GDM recommend a minimum of 175 g of carbohydrate per day and provide recommendations for meal frequency and content, but they lack specific guidance on meal timing in relation to sleep–wake cycles [34]. These guidelines typically advise distributing carbohydrate intake across three small-to-moderate sized meals and two or more snacks throughout the day. However, they do not address the potential benefits of aligning meal times with circadian rhythms, such as considering the timing of breakfast relative to waking or the interval between the last meal and sleep onset. Nevertheless, current evidence supports the notion that pregnancy is associated with profound changes in the levels of glucoregulatory hormones, contributing to alterations in glycemic control, insulin sensitivity, and insulin secretion. While most glucoregulatory hormones (e.g., prolactin, cortisol, glucagon, melatonin) maintain their pre-pregnancy circadian/diurnal rhythmicity, others (e.g., growth hormone) become non-rhythmic, adding complexity to metabolic regulation during pregnancy [35]. This gap in the guidelines represents an opportunity to incorporate emerging evidence on chrono-nutrition into GDM management strategies, potentially enhancing glycemic control and improving maternal and fetal outcomes.

Poor sleep was reported in 45% of women in our cohort, excessive daytime sleepiness in 53%, and snoring in 22%. Previous studies conducted in both healthy women and women with GDM demonstrated an association between sleep disorders and increased risk of maternal hyperglycemia and adverse obstetrical outcomes, particularly among women with pre-pregnancy obesity [36,37]. This oversight is particularly concerning given that sleep disturbances may exacerbate insulin resistance and contribute to poor glycemic control during pregnancy. Recent research by Weschenfelder et al. on circadian rhythms and GDM highlights the importance of considering working conditions, sleeping habits, and lifestyle factors in relation to insulin dependency during pregnancy [38]. Despite the growing literature linking sleep disorders and glycemic control in pregnant women, sleep is rarely addressed in the guidelines for pregnant women with GDM [34]. The majority of women in our cohort were overweight or obese before pregnancy. Almost 18% of participants reported gestational weight gain above recommendations. These characteristics of obesity, together with the high rate of poor sleep quality, late chronotype, and late night-time eating, suggest that these chronobiological factors may be linked.

The strengths of our study include the prospective design, single-center setting, and heterogeneous population with high adherence. In addition, we performed face-to-face structured interviews with a trained investigator. Study limitations include the use of self-reported data on nutrition and sleep and self-monitoring of blood sugar measurements, which can lead to inaccuracies (but not to misclassification bias).

## 5. Conclusions

The findings of this unique prospective cohort study investigating multiple chronobiological factors in women with GDM revealed that dietary content and timing of food consumption in addition to sleep quality are associated with glycemic control and neonatal birth weight. The results point to the important role of chrono-nutrition and sleep in the management of women with GDM. Future studies are needed to develop and evaluate interventions that address these chronobiological factors to improve outcomes for both mothers and infants in GDM pregnancies.

## Figures and Tables

**Table 1:** Demographic, anthropometric, and glycemic characteristics at baseline in 208 women with gestational diabetes mellitus.

Parameters	Value
Demographic data	
Maternal age (y)	33.2 ± 5.1
Maternal age distribution (y)	
25–18	16 (7.7)
30–26	38 (18.3)
35–31	78 (37.5)
40–36	60 (28.8)
>40	16 (7.7)
Education (y)	
<12	30 (14.4)
≥12	178 (85.6)
Marital status	
Married/in partnership	189 (90.9)
Single mother	19 (9.1)
Religiosity	
Ultra-religious/religious	49 (23.5)
Traditional/secular	159 (76.5)
Number of children	
0	72 (34.6)
1–2	107 (51.4)
3–4	22 (10.6)
5+	7 (3.4)
Anthropometric data	
Pre-pregnancy weight (kg)	72.3 ± 18.8
Pre-pregnancy BMI distribution (kg/m^2^)	
Underweight (<18.5 kg/m^2^)	7 (3.4)
Normal weight (18.5–24.9 kg/m^2^)	78 (37.5)
Overweight (25.0–29.9 kg/m^2^)	61 (29.3)
Obese (≥30.0 kg/m^2^)	62 (29.8)
Glycemic background	
First-degree relative with type 2 diabetes	81 (38.9)
GDM in previous pregnancies (For women who have given birth, *n* = 136)	45 (33.0)
Previous LGA newborn (Prima/multi gravida, *n* = 136)	51 (37.5)

Numeric scales are reported as mean ± standard deviation, and categorical measures are reported as frequency (percent). BMI, body mass index; GDM, gestational diabetes mellitus; LGA, large for gestational age.

**Table 2:** Lifestyle, sleep, and chronobiological characteristics in 208 women with gestational diabetes mellitus.

Characteristics	Value
Smoker	
Prior to pregnancy	36 (17.3)
Current	11 (5.3)
Physical activity	
Current physical activity	44 (21.2)
Coffee consumption (cups per day)	
0	53 (25.5)
1–2	125 (60.0)
3–4	29 (14.0)
>4	1 (0.5)
Coffee consumption after 5 pm	18 (11.6)
Sleep assessment	
Sleep quality (PSQI score)	
Good (score 0–5)	114 (54.8)
Poor (score 6–21)	94 (45.2)
Daytime sleepiness (ESS score)	
No daytime sleepiness (score 1–10)	98 (47.1)
Daytime sleepiness (score 11–24)	110 (52.9)
Snoring	
Snoring before pregnancy	19 (9.1)
Current snoring GW 24–28	46 (22.1)
Chronotype	
Morning type	149 (71.6)
Evening type	59 (28.4)

Numeric scales are reported as mean  ±  standard deviation, and categorical measures are reported as frequency (percent). PSQI, Pittsburgh Sleep Quality Index; ESS, Epworth Sleepiness Scale.

**Table 3:** Chrono-nutrition characteristics in 208 women with gestational diabetes mellitus.

Parameters	Value
Total per day	
Calories (kcal)	1993.6 ± 385.12
Carbohydrates (g)	178.97 ± 40.57
Proteins (g)	96.94 ± 27.84
Fats (g)	98.88 ± 28.02
From wake-up to 12 p.m. (absolute values)	
Calories (kcal)	367.70 ± 167.29
Carbohydrates (g)	42.39 ± 18.25
Proteins (g)	16.24 ± 8.28
Fats (g)	14.79 ± 10.91
From wake-up to 12 p.m. (percent of total calories)	
Carbohydrates	48 ± 12
Proteins	18 ± 5
Fats	33 ± 13
From 12 p.m. to 6 p.m. (absolute values)	
Calories 12 p.m. to 6 p.m. (kcal)	1114.57 ± 234.41
Carbohydrates (g)	90.59 ± 26.56
Proteins (g)	54.84 ± 18.67
Fats (g)	59.20 ± 19.94
From 12 p.m. to 6 p.m. (percent of total calories)	
Carbohydrates	33 ± 10
Proteins	19 ± 6
Fats	46 ± 9
From 6 p.m. to bedtime (total calories)	
Calories (kcal)	511.31 ± 221.16
Carbohydrates (g)	45.99 ± 23.42
Proteins (g)	25.85 ± 16.75
Fats (g)	24.88 ± 14.27
From 6 p.m. to bedtime (percent of total calories)	
Carbohydrates	36 ± 12
Proteins	20 ± 8
Fats	42 ± 12
Interval between wake-up time and breakfast (minutes)	
≤30	51 (24.5)
30.1–60	49 (23.6)
60.1–120	36 (17.3)
>120	72 (34.6)
Interval between last meal and bedtime (minutes)	
≤30	36 (17.3)
30.1–60	31 (14.9)
60.1–120	35 (16.8)
>120	106 (51.0)

Numeric scales are reported as mean  ±  standard deviation.

**Table 4:** Glycemic, anthropometric, and obstetric characteristics at the end of follow-up in 208 women with gestational diabetes mellitus.

Parameters	Value
Gestational weight gain by IOM recommendation, *n* = 201	
Below recommended value	67 (33.3)
In accordance with recommended value	98 (48.8)
Above recommended value	36 (17.9)
Treatment for glycemic balance	
Nutritional	108 (51.9)
Nutritional + oral drug therapy	51 (24.5)
Nutritional + insulin therapy	43 (20.7)
Nutritional + oral drug therapy + insulin therapy	6 (2.9)
Level of glycemic control	
Suboptimal	89 (42.7)
Optimal	119 (57.3)
Neonatal gender	
Male	102 (49.0)
Female	106 (51.0)
Mode of delivery	
Vaginal	145 (69.7)
Instrumental	53 (25.5)
Cesarean section	10 (4.8)
Gestational age at birth (weeks)	38.3 ± 1.4
Gestational age at birth distribution (weeks)	
24–34	1 (0.5)
34–37	6 (2.9)
>37	201 (96.6)
Neonatal birth weight (g)	3194.8 ± 408.1
Neonatal birth-weight distribution (g)	
<2500	14 (6.7)
2500–3999	193 (92.8)
4000+	1 (0.48)
Birth weight percentile	58.1 ± 25.77
Birth weight percentile categories	
Below 85th percentile	168 (80.8)
At or above 85th percentile	40 (19.2)

Numeric scales are reported as mean  ±  standard deviation, and categorical measures are reported as frequency (percent). IOM, Institute of Medicine.

**Table 5:** Relative risk for suboptimal maternal glycemic control in 208 women with gestational diabetes.

Parameter	RR	95% CI		*p* Value
Maternal age	1.04	0.96	1.12	0.3
Number of children	0.96	0.75	1.24	0.79
Past birth-weight percentile				0.67
Below 85th percentile	1			
At or above 85th percentile	0.83	0.35	1.96	
Pre-pregnancy BMI	0.97	0.92	1.03	0.39
Treatment for glycemic balance				<0.001
Nutritional	1			
Nutritional + oral drug therapy and/or insulin therapy	4.11	2.62	6.45	
Interval from wake-up to breakfast				0.02
30 min or less	1			
More than 30 min	2.26	1.09	4.67	
Carbohydrate intake from wake-up to 12 p.m. (increase of 10 g)	1.02	0.84	1.25	0.77
Carbohydrate intake from 6 p.m. to bedtime (increase of 10 g)	1.19	1.003	1.42	0.04
Sleep quality (PSQI score)				0.03
Good (score 0–5)	1			
Poor (score 6–21)	2.14	1.04	4.41	

RR, relative risk; 95% CI, confidence interval; PSQI, Pittsburgh Sleep Quality Index.

**Table 6:** Relative risk for birth-weight percentile for gestational age at or above the 85th percentile in 208 women with gestational diabetes.

Parameter	RR	95% CI		*p* Value
Maternal age	1.06	0.96	1.17	0.24
Number of children	1.19	0.86	1.63	0.27
Past birth-weight percentile				0.001
Below 85th percentile	1			
At or above 85th percentile	5.36	1.92	14.92	
Treatment for glycemic balance				0.8
Nutritional	1			
Nutritional + oral drug therapy and/or insulin therapy	0.93	0.53	1.62	
Gestational weight gain				0.53
Below/in accordance with IOM recommendation	1			
Above IOM recommendation	1.23	0.63	2.42	
Carbohydrate intake from wake-up to 12 p.m. (increase of 10 g)	1.7	1.3	2.23	<0.001
Carbohydrate intake from 6 p.m. to bedtime (increase of 10 g)	1.39	1.16	1.67	<0.001
Sleep quality (PSQI score)				0.19
Good (score 0–5)	1			
Poor (score 6–21)	0.52	0.19	14.92	
Level of glycemic control				0.04
Optimal	1			
Suboptimal	2.85	1.02	7.95	

RR, relative risk; 95% CI, confidence interval; PSQI, Pittsburgh Sleep Quality Index.

## Data Availability

The data that support the findings of this study are available from the corresponding author, upon reasonable request.
